# Factors related to Nursing workload in the Oncology assistance provided to hospitalized women[Fn fn1]

**DOI:** 10.1590/1518-8345.6787.4107

**Published:** 2024-03-15

**Authors:** Talita Balaminut, Gabriela Alves Godoy, Elenice Valentim Carmona, Ariane Polidoro Dini

**Affiliations:** 1 Universidade Estadual de Campinas, Faculdade de Enfermagem, Campinas, SP, Brazil.; 2 Scholarship holder at the Conselho Nacional de Desenvolvimento Científico e Tecnológico (CNPq), Brazil.

**Keywords:** Workload, Occupational Health, Nursing Team, Hospital Oncology Service, Oncology Nursing, Women’s Health, Carga de Trabajo, Salud Laboral, Grupo de Enfermería, Servicio de Oncología en Hospital, Enfermería Oncológica, Salud de la Mujer, Carga de Trabalho, Saúde Ocupacional, Equipe de Enfermagem, Serviço Hospitalar de Oncologia, Enfermagem Oncológica, Saúde da Mulher

## Abstract

**Objective:**

to evaluate the Nursing workload and its related factors in the assistance provided to hospitalized women with gynecological and breast cancers, according to the Nursing Activities Scores adapted for cancer patients.

**Method:**

a cross-sectional and epidemiological study. The participants were women with gynecological and/or breast cancer, over 18 years of age, and hospitalized for a minimum period of 24 hours. The following was collected from the medical records: sociodemographic and clinical data, Karnofsky Performance Status and workload, according to the adapted Nursing Activities Score. The factors related to workload were analyzed by means of multiple linear regression.

**Results:**

the mean Nursing Activities Scores was 29.3%, denoting seven hours of daily care per patient. The factors related to workload differed according to the breast or gynecological cancer diagnosis (β=-0.01; p<0.001), clinical or surgical treatment (β=-0.03; p<0.001) and functional capacity at admission (β=0.07; p<0.001), as per the Karnofsky Performance Status.

**Conclusion:**

there was greater workload for the care of women with gynecological cancer undergoing clinical treatment and with lower functional capacity at admission. The findings reveal directions for optimization of resources and improvements in work processes and flows, in order to promote a favorable work environment and good quality assistance.

## Resumen

**Objetivo:**

evaluar la carga de trabajo de enfermería y los factores relacionados con la atención de mujeres hospitalizadas con cáncer ginecológico y de mama, según el *Nursing Activities Scores* adaptado para pacientes oncológicos.

**Método:**

estudio epidemiológico y transversal. Participaron mujeres con cáncer ginecológico y/o de mama, mayores de 18 años, hospitalizadas por un período mínimo de 24 horas. De las historias clínicas se recolectaron datos sociodemográficos y clínicos, *Karnofsky Performance Status* y carga de trabajo, según el *Nursing Activity Score* adaptado. Los factores relacionados con la carga de trabajo se analizaron mediante regresión lineal múltiple.

**Resultados:**

el puntaje promedio del *Nursing Activity Scores* fue del 29,3%, lo que indica que se brindan siete horas diarias de atención por paciente. Los factores relacionados con la carga de trabajo difirieron según diagnóstico de cáncer de mama o ginecológico (β=-0,01; p<0,001), tratamiento clínico o quirúrgico (β=-0,03; p<0,001) y capacidad funcional al momento del ingreso (β=0,07; p< 0,001), conforme a la escala *Karnofsky Performance Status* .

**Conclusión:**

hubo una mayor carga de trabajo en la atención a mujeres con cáncer ginecológico en tratamiento clínico y con menor capacidad funcional al momento del ingreso. Los hallazgos revelan información útil para optimizar recursos, mejorar procesos y flujos de trabajo, con el fin de promover un ambiente de trabajo favorable y una atención de calidad.

## Resumo

**Objetivo:**

avaliar a carga de trabalho da enfermagem e seus fatores relacionados na assistência às mulheres hospitalizadas com cânceres ginecológicos e mamários, segundo o *Nursing Activities Scores* , adaptado a pacientes oncológicos.

**Método:**

estudo epidemiológico de corte transversal. Participaram mulheres com câncer ginecológico e/ou mamário, maiores de 18 anos, hospitalizadas por período mínimo de 24 horas. Coletados, do prontuário, dados sociodemográficos, clínicos, *Karnofsky Performance Status* e carga de trabalho, segundo *Nursing Activities Score* adaptado. Os fatores relacionados à carga de trabalho foram analisados por regressão linear múltipla.

**Resultados:**

pontuação média do *Nursing Activities Scores* foi 29,3%, denotando sete horas de assistência diária por paciente. Os fatores relacionados à carga de trabalho diferiram conforme diagnóstico de câncer de mama ou ginecológico (β= - 0,01; p<0,001), tratamento clínico ou cirúrgico (β= - 0,03; p<0,001) e capacidade funcional na admissão (β= 0,07; p<0,001), pelo *Karnofsky Performance Status* .

**Conclusão:**

evidenciou-se maior carga de trabalho para atendimento de mulheres com câncer ginecológico sob tratamento clínico e com menor capacidade funcional na admissão. Os achados revelam direcionamentos para otimização de recursos, melhorias em processos e fluxos de trabalho, a fim de promover ambiente laboral favorável e assistência de qualidade.

**Highlights:**

**(1)** A pioneer study in using the NAS instrument, adapted for Hospital Oncology.

**(2)** The clinical treatment of gynecological/breast cancer demands a greater workload.

**(3)** Functional capacity at the admission of women with cancer influences workload.

**(4)** Gynecological cancer demands a greater Nursing workload than breast cancer.

## Introduction

 The care of people with cancer is challenging and highly complex, including the management of symptoms arising from evolution of the disease or from intensive and increasingly complex treatments, which increases the demand for Nursing care ^(^ ^1^ ^-^ ^2^ ^)^ . 

 In cases of breast and gynecological cancers, there are singularities involved in the illness, especially related to subjective aspects and the repercussions of the disease on women’s lives, such as possible mutilations and impacts on self-image and sexuality ^(^ ^3^ ^-^ ^4^ ^)^ . Thus, the performance of the Nursing team in the care of this clientele needs to include, in addition to physical, functional and therapeutic issues, emotional, sociocultural, self-image and sexual functioning needs, extended to family members and spouses, in an integrated way ^(^ ^4^ ^-^ ^5^ ^)^ . 

 In this context, the Nursing work environment is frequently permeated by stress and pressure, related to decision-making and constant interactions with patients and their families, in situations with potential for emotional exhaustion ^(^ ^6^ ^)^ . In addition, frequent exposure to risks, occupational stress, physical exhaustion and insufficient human and material resources also increases the physical and psychological burden of the Oncology Nursing team, oftentimes characterizing work overload ^(^ ^2^ ^,^ ^7^ ^)^ . 

 The analysis of the Nursing workload, in the most diverse contexts, is indispensable due to the complexity of issues in the work environment that interfere with workers’ health and with patient care safety ^(^ ^7^ ^)^ . Nursing work overload can configure risk conditions for patients, families and professionals alike ^(^ ^7^ ^)^ , trigger care omissions ^(^ ^8^ ^)^ , and be related to mortality, hospital readmission and longer hospitalization times ^(^ ^9^ ^)^ . For the professionals, the workload can be related to job satisfaction, to the workers’ feeling that their role is important, significant and worthwhile. When excessive, it can influence the acute and chronic fatigue levels ^(^ ^10^ ^)^ and trigger Burnout ^(^ ^6^ ^-^ ^11^ ^)^ , as well as increase absenteeism in Nursing ^(^ ^12^ ^)^ , also showing the relevance of its monitoring. 

 Evaluating the Nursing workload subsidizes the management of human and material resources, adequate staffing, and a reduction in costs by preventing adverse events and waste. In addition, it helps the fair division of labor and the favorable environment for the professionals’ health, promoting improvements in care safety and quality, with reduced harms to the patients ^(^ ^9^ ^,^ ^13^ ^-^ ^14^ ^)^ . 

 Several tools were developed to estimate the workload for the evaluation of Nursing activities, among them the Nursing Activities Score (NAS), which was translated and validated for Brazilian Portuguese ^(^ ^15^ ^)^ . Originally developed for the direct measurement of workload in intensive care units, its use was expanded to other sectors ^(^ ^13^ ^)^ . In 2018, a number of researchers adapted the content of NAS to Nursing care in Hospital Oncology ^(^ ^16^ ^)^ . 

 Although the original version of NAS ^(^ ^15^ ^)^ has been used in intensive care units, the content adaptation to the Oncology context ^(^ ^16^ ^)^ was not applied to this type of scenario, and this study was pioneering. Moreover, there is scarcity of studies that help determine the workload of the Nursing team in the Hospital Oncology sector ^(^ ^17^ ^)^ , especially in Gynecological and Breast Oncology. Thus, little is known about the Nursing care demands required by hospitalized women with breast and/or gynecological cancer and undergoing clinical and/or surgical treatments, according to NAS, as well as the factors related to workload in this context. These are gaps that this study aims at bridging. 

Therefore, it becomes fundamental to identify the activities that require more care time for women with gynecological and breast cancers; as well as to recognize the factors related to the women’s sociodemographic and clinical characteristics that may overwhelm the Nursing team, in order to equip health services for the development of proposals for adequate and safe working conditions for the professionals, as well as good quality care for women. Thus, this study aimed at evaluating the Nursing workload and its related factors in the assistance provided to hospitalized women with gynecological and breast cancers, according to NAS, adapted to cancer patients.

## Method

### Study design

This is a quantitative, cross-sectional and analytical study guided by the Strengthening the Reporting of Observational Studies in Epidemiology (STROBE) tool, recommended by the Enhancing the Quality and Transparency of Health Research Network (EQUATOR Network).

### Locus

The study was carried out at a public teaching hospital, specialized in women’s care and located in Campinas, in the inland of the state of São Paulo, Brazil, which assists patients from the Unified Health System in the Obstetrics, Gynecology and Oncology specialties. The data were collected in inpatient Oncology units devoted to assisting women with gynecological and breast neoplasms, subdivided into Clinical Oncology (15 beds) and Surgical Oncology (20 beds).

### Period

Data collection was carried out between October 2021 and January 2022.

### Participants

Women with gynecological and/or breast cancers, admitted to the Clinical and Surgical Oncology units of the aforementioned hospital.

### Selection criteria

The inclusion criteria were as follows: women aged at least 18 years old, admitted to one of the two inpatient cancer units for a minimum of twenty-four hours and diagnosed with gynecological and/or breast cancer. Women readmitted during the collection period were included in the sample and considered new participants.

### Definition of the sample

The convenience sample consisted of women admitted to the Oncology units according to the inclusion criteria. Due to convenience sampling, sample calculation was not considered.

### Data collection instruments and study variables

The data were collected through a questionnaire to characterize the participants and the NAS instrument, adapted to Oncology.

The characterization questionnaire was prepared by the first author and submitted to a pilot test with five eligible women, proving to be adequate. The five participants in the pilot test were excluded from the final sample. The questionnaire included the following variables: age, marital status, hospitalization date, origin, cancer diagnosis, date of the first cancer diagnosis, type of treatment, presence of comorbidities and metastases, previous surgery, chemotherapy and radiotherapy during hospitalization, presence of a companion during hospitalization, functional capacity to perform common tasks according to the Karnofsky Performance Status (KPS) scale at admission, and hospitalization time and outcome.

 The KPS scale assesses cancer patients’ clinical performance and decline through their ability to continue their usual activities and jobs. It also assesses their need for support or their dependence on constant care in order to continue living ^(^ ^18^ ^)^ . The result of this scale varies from 0% to 100%, with 0% representing death, whereas 100% means preservation of functional capacity and absence of evidence of the disease and symptoms ^(^ ^18^ ^)^ . 

 NAS is a stable measuring instrument to assess Nursing workload, covering 80.8% of the activities ^(^ ^15^ ^-^ ^16^ ^)^ . Despite exceeding the scope of other instruments that also analyze the time spent by Nursing in patient care ^(^ ^15^ ^)^ , NAS fails to take into account some relevant activities, such as preceptorship of Nursing students, training of new members of the clinical and non-clinical team, and time to learn new initiatives, changes in requirements, documentation and patient assessment instruments ^(^ ^19^ ^-^ ^20^ ^)^ . 

 On the other hand, both the original version of NAS and the one adapted to Oncology include bureaucratic and managerial aspects. In addition, it offers the analysis of the need for Nursing support for patients and their families in relation to counseling, dealing with pain, anguish, difficult family circumstances, communication of bad news, anxiety, fear of death, expectations of family members, palliative care and care in the final stage of life ^(^ ^15^ ^-^ ^16^ ^)^ . These activities are very present in the context to be studied, justifying the choice of the adapted NAS instrument. 

 Both NAS versions consist of 23 items subdivided into seven categories (basic activities, ventilatory support, cardiovascular support, renal support, neurological support, metabolic support and specific interventions), which represent care measures and evaluations to which values from 1.2 to 32 are assigned. The NAS score, whose mean value was the dependent variable of this study, represents how much time (in percentage) of work the patient required in the last 24 hours. If this score exceeds 100%, it means that the patient required more than one Nursing professional for assistance in a given work shift. Each NAS point equals 14.4 minutes or 0.24 hours of assistance. The maximum NAS score is 176.8% ^(^ ^15^ ^-^ ^16^ ^)^ . 

 The adaptation of the NAS content to make it feasible and reliable for the care of cancer patients sought to maintain the structure of the original version, as well as the items, order and score ^(^ ^16^ ^)^ , using adapted version for collection. Although this adapted instrument is in the public domain, authorization was obtained from the authors for its use in this research. 

### Data collection

Data collection was performed by the first and second authors and by a research assistant, an undergraduate Nursing student. All were previously trained to apply the instruments.

 The women were invited to participate in the research and, after accepting and signing the Informed Consent Form, the data were collected during the participants’ hospitalization. The characterization data were collected from the physical and/or electronic medical records, as well as the NAS application adapted to cancer patients ^(^ ^16^ ^)^ , which took place daily until the hospitalization in Oncology units outcome. The data were collected from the medical records because they are the easiest place to access all the information required to complete NAS. When necessary, some data were collected or confirmed with the woman/companion, or with the nurses in the unit. 

Applied daily throughout the women’s hospitalization, NAS covered the last 24 hours of assistance, considering the period from 7 am to 7 am the following day. On the first hospitalization day, the activities were considered from the time of admission to the unit until 7 am the following day. On the hospitalization outcome day, the activities were evaluated from 7 am until the moment of leaving the sector.

 To support completion of NAS, a manual was used to guide its application and standardize the meaning of each of its items, in order to avoid possible misinterpretation ^(^ ^14^ ^)^ , in addition to the guidelines and descriptions found in the version adapted to cancer patients ^(^ ^16^ ^)^ . 

### Data treatment and analysis

The data were organized in an electronic spreadsheet, using Excel for Windows. Independent double typing and verification of errors and inconsistencies were performed. Subsequently, the data were transferred and analyzed using the Statistical Analysis System (SAS) software, version 9.4.

The workload was obtained by adding the NAS points of each woman in the sample. The mean daily score for all women and the period under study was calculated. In addition, the NAS scores on the days corresponding to admission and outcome of the participants’ hospitalization were analyzed. These data and the frequency of the NAS items were analyzed using absolute and relative frequency distribution.

The qualitative and quantitative variables were described by calculating frequencies and percentages. Measures of central tendency and dispersion were also described. For the comparisons of the sociodemographic and clinical variables to the mean NAS score, the Mann-Whitney test was performed for the qualitative variables and Spearman’s correlation coefficient for the quantitative ones. The association analyses between the dichotomized KPS scale and the “presence of metastasis” and “cancer diagnosis” variables were performed using Pearson’s Chi-square test. The data distributions of the mean NAS score and hospitalization time of the women were evaluated using the Shapiro-Wilk test, analyzed by SAS.

 A multiple linear regression model was constructed, via generalized linear models, considering the NAS score as the dependent variable and the sociodemographic and clinical variables that had p-values < 0.20 in the comparison and correlation analyses as independent variables. The model was adjusted considering Normal distribution and the Identity link function. To meet the distribution assumption, the Box-Cox transformation ^(^ ^21^ ^)^ was applied to the dependent variable data. The transformation suggested by the Box-Cox method was the inverse of the square root of the dependent variable. In this model, the estimates of the regression coefficients were presented, as well as their confidence intervals and p-values. A 5% significance level was considered for all analyses. 

### Ethical aspects

The study was evaluated and approved by the local Research Ethics Committee, under Certificate of Presentation of Ethical Appraisal No. 49160821.3.0000.5404 and opinion No. 4,910,826/2021. All ethical precepts established in Resolution No. 466/2012 of the National Health Council were respected.

## Results

The participants were 231 women hospitalized in Oncology units, with a mean age of 54.3 (±13.5) years old. 56.7% (n=131) reported having a partner and were hospitalized for a mean of 3.7 (±3.9) days, with a minimum of one and a maximum of 30 days. 59.7% (n=138) of them were hospitalized from their homes, followed by 12.1% (n=28) from outpatient clinics, 11.7% (n=27) from emergency care services, 8.2% (n=19) from intensive care units, 5.6% (n=13) from other sectors of the hospital and 2.6% (n=6) from another hospital. Of the participants, 14.7% (n=34) were readmitted to the aforementioned units during the four months of the study data collection period.

More than half of the women (63.2%; n=146) had been diagnosed with cancer at least one year ago. Among the participants, 116 (50.2%) had breast cancer and 115 (49.8%) had gynecological cancer, with endometrial (16.0%) and cervical (15.2%) and ovary (8.7%) tumors as the most prevalent. In this hospitalization, 62.8% (n=145) of the women were admitted for surgical treatments and 37.2% (n=86) for clinical treatments. There was only one hospitalization for emergency surgery and 16 (6.9%) women were in palliative care.

 Of the 135 women who presented some comorbidity (58.4%), the most prevalent ones were systemic arterial hypertension, diabetes *mellitus* , thyroid disorders (hypothyroidism and hyperthyroidism), dyslipidemia, depression and nephropathies or renal failure. 26.4% (n=61) of the women had metastases, with 45.9% (n=28) of them in the lung/pleura region, 34.4% (n=21) in bones, 34.4% (n=21) in the lymphatic system/lymph nodes, 32.8% (n=20) in the liver and 16.4% (n=10) in the Central Nervous System, considering that some women had metastasis in more than one location. 

In relation to the functional capacity at admission, 77.9% (n=180) had KPS scores above 50%, indicating capacity for self-care and activities without the need for special care. 22.0% (n=51) of the women had KPS scores of 50% or less, indicating inability, limited self-care autonomy and the need for greater care, including hospital assistance. Of the women without metastases, 81.7% (n=139) had KPS scores above 50%, at the same time that, of those who had metastases, 67.2% (n=41) had KPS scores above 50% (p=0.0187). Of the women with breast cancer, 84.4% (n=98) had KPS values above 50%, whereas of those with gynecological cancer, 71.3% (n=82) had KPS scores above 50% (p=0.0158).

The majority (77.1%; n=178) also did not require a companion during hospitalization. Only 4.8% (n=11) of the women received chemotherapy and 2.2% (n=5) underwent radiotherapy during hospitalization.

Regarding the hospitalization outcome, 84.4% (n=195) were discharged home and 13.0% (n=30) were transferred to another sector or hospital, including an intensive care unit, while 2.6% (n=6) evolved to death. All patients who died during the research were undergoing clinical treatments, five of them under palliative care.

 The NAS score and the Nursing care hours according to this adapted instrument are presented in Table 1 . 

**Table 1:** - Distribution of the mean Nursing Activities Score, on the days corresponding to admission and to the hospitalization outcome of women hospitalized with gynecological and breast cancer, according to their score and Nursing care hours (n ^*^ =231). Campinas, SP, Brazil, 2021-2022

NAS ^†^ variables	Mean (SD ^‡^ )	Median	Minimum-Maximum
Mean NAS ^†^ score (%)	29.3 (±11.4)	25.9	12.8-82.2
Mean Nursing hours (hours)	7.0 (±2.7)	6.2	3.1-19.7
NAS ^†^ score at admission (%)	29.9 (±15.0)	23.9	12.8-87.2
Nursing hours at admission (hours)	7.2 (±3.6)	5.7	3.1-20.9
NAS ^†^ score at hospitalization outcome (%)	28.4 (±14.9)	23.9	12.8-102.3
Nursing hours at outcome (hours)	6.8 (±3.6)	5.7	3.1-24.6

* n = Sample size;

† NAS = Nursing Activities Score;

‡ SD = Standard Deviation

When evaluating the 23 types of Nursing interventions found in the NAS instrument, those that obtained a higher mean percentage of scored days were as follows: monitoring and controls (100% of the days); administrative and managerial tasks (99.5% of the days; ±4.1); hygiene procedures (98.8% of the days; ±8.0); medication (91.9% of the days; ±20.7); and specific interventions outside the unit (23.5% of the days; ±18.1). The following items were not scored in all patients: left atrium monitoring; cardiorespiratory resuscitation; and intracranial pressure measurements.

The activities that required the longest mean time from the Nursing team in the 24 hours for each of the hospitalized women were the following: monitoring and controls (1 hour and 20 minutes); hygiene procedures (1 hour and 18 minutes); administrative and managerial tasks (1 hour and 17 minutes); and medications (1 hour and 14 minutes).

 The relationship of the mean NAS score of the women hospitalized in the Oncology units with their sociodemographic characteristics and clinical aspects is presented in Table 2 . 

**Table 2:** - Mean Nursing Activities Score, according to sociodemographic and clinical variables of women hospitalized with gynecological and breast cancers (n ^*^ =231). Campinas, SP, Brazil, 2021-2022

Variables	Mean Nursing Activities Score					
	n*	Mean (SD ^†^ )	Median	Minimum-Maximum	p-value ^‡^ (Distribution)	p-value ^§^ (Comparison)
Age group (years old)						
<60	152	28.9 (10.3)	26.2	13.7-70.3	<0.0001	0.9512
>60	79	30.0 (13.4)	25.6	12.8-82.1	<0.0001	
Marital status						
With a partner	131	28.1 (10.2)	24.7	12.8-69.6	<0.0001	0.0786
Without a partner	100	30.8 (12.8)	27.7	13.7-82.1	<0.0001	
Cancer diagnosis						
Breast cancer	116	26.8 (9.7)	24.3	12.8-79.6	<0.0001	0.0016
Gynecological cancer	115	31.7 (12.5)	28.7	15.9-82.1	<0.0001	
Time since cancer diagnosis (years)						
<1	85	29.0 (10.9)	25.3	13.7-79.6	<0.0001	0.9837
>1	146	29.4 (11.8)	26.2	12.8-82.1	<0.0001	
Type of treatment						
Clinical	86	36.0 (13.6)	33.2	18.7-82.1	<0.0001	<0.0001
Surgical	145	25.2 (7.6)	23.3	12.8-54.0	<0.0001	
Presence of comorbidities						
Yes	135	29.7 (11.9)	25.8	12.8-82.1	<0.0001	0.4888
No	96	28.6 (10.7)	26.5	15.9-79.6	<0.0001	
Previous surgery						
Yes	124	29.1 (11.3)	26.0	12.8-70.3	<0.0001	0.5988
No	107	29.5 (11.6)	25.9	13.7-82.1	<0.0001	
Metastasis						
Yes	61	31.7 (10.1)	29.9	18.7-60.7	<0.0001	0.0027
No	170	28.4 (11.8)	24.5	12.8-82.1	0.0008	
Karnofsky Performance Scale at admission						
≤50%	51	42.8 (13.7)	40.7	24.7-82.1	0.0013	<0.0001
>50%	180	25.4 (7.0)	23.5	12.8-50.5	<0.0001	
Readmission						
Yes	34	35.3 (12.6)	35.7	12.8-70.3	<0.0001	0.0005
No	197	28.2 (10.9)	24.7	13.7-82.1	0.2786	

* n = Sample size;

† SD = Standard Deviation;

‡ p-value = Obtained by means of the Shapiro-Wilk test;

§ p-value = Obtained by means of the Mann-Whitney test

The Shapiro-Wilk test applied to the “hospitalization time” variable indicated that the data did not follow Normal distribution (p<0.0001).

The mean NAS score presented a moderate positive correlation (0.36; p<0.0001) with the women’s hospitalization days. The women under palliative care had a mean NAS score of 45.3% (±17.1).

 The women with gynecological cancer had a difference in the median NAS score 4.4 points higher, equivalent to one hour and 34 minutes, than those with breast cancer. The participants in clinical treatments had a median of 9.9 NAS points higher, that is, two hours and 23 minutes more in Nursing care, compared to those undergoing surgical treatments. The women with KPS ≤ 50% at admission had a median of 17.2 NAS points higher, which equals 4 hours and 8 minutes, than those with KPS > 50% ( Table 2 ). These relationships were maintained in the multiple linear regression analysis, as shown in Table 3 . 

**Table 3:** - Linear regression of the mean Nursing Activities Score with sociodemographic and clinical variables of hospitalized women with gynecological and/or breast cancer (n ^*^ =231). Campinas, SP, Brazil, 2021-2022

Variables	Linear Regression ^†^		
	Coefficient (β)	LL ^‡^ ; UL ^§^	p-value ^||^
Marital status			
With a partner	0.00	-0.01; 0.01	0.9087
Without a partner	Reference		
Cancer diagnosis			
Breast cancer	-0.01	-0.03; - 0.01	0.0258
Gynecological cancer	Reference		
Hospitalization time (days)	0.00	0.00; 0.00	0.2193
Type of treatment			
Surgical	-0.03	-0.05; -0.02	<0.0001
Clinical	Reference		
Metastasis			
No	-0.01	-0.02; 0.01	0.3523
Yes	Reference		
Karnofsky Performance Scale at admission			
≤50%	0.07	0.05; 0.09	<0.0001
>50%	Reference		
Readmission			
No	-0.01	-0.03; 0.01	0.3567
Yes	Reference		

* n = Sample size;

† The Box-Cox transformation was applied to the dependent variable;

‡ LL = Lower Limit;

§ UL = Upper Limit;

|| p-value = Significance Level

The women with breast cancer had a decreased NAS score and, therefore, shorter Nursing care time, (β=-0.01; p=0.0258) when compared to those with gynecological cancer, as well as women undergoing surgical treatments presented a decreased NAS score (β=-0.03; p<0.0001) when compared to those undergoing clinical treatments. In addition, the participants who presented KPS at admission less than or equal to 50%, indicating less autonomy for self-care, representing an increase in the NAS score (β=0.07; p<0.0001); in other words, they required longer Nursing care time per day when compared to those with KPS at admission above 50%.

By estimating the regression equation, considering the “Hospitalization time” variable equal to zero and the other independent variables, presenting as results their respective reference categories, it was possible to identify that, for women with breast cancer, the predicted NAS value was 27.45 points and. for those with gynecological cancer, it was 29.60 points; that is, women with breast cancer had a mean of 2.15 NAS points (31 minutes) less than those with gynecological cancer.

For women undergoing surgical treatments, the predicted NAS value was 25.14 points and, for those on clinical treatments, it was 29.60 points. Thus, women undergoing surgical treatments had a mean of 4.46 NAS points less, receiving a mean of one hour and four minutes less Nursing care than those undergoing clinical treatments.

For women with KPS at admission less than or equal to 50%, the predicted NAS value was 44.92 points and, for those who had KPS above 50%, the value was 29.60 points. Therefore, women with KPS at admission less than or equal to 50% had a mean of 15.32 points more than those with KPS above 50%, equivalent to three hours and 41 minutes more of Nursing care.

## Discussion

The women with gynecological and/or breast cancer in inpatient Oncology units presented a mean of seven hours of Nursing care in 24 hours. The factors associated with the Nursing team workload were the cancer diagnosis, the type of treatment to which the women were being submitted at their hospitalization and their functional capacity at admission, according to KPS.

 Although NAS does not categorize patients into complexity levels, it is possible to transform its score into time spent, enabling this analysis ^(^ ^20^ ^)^ according to Federal Nursing Council Resolution No. 543/2017 ^(^ ^22^ ^)^ . According to this Resolution, patients who demand up to six hours of daily Nursing are considered as in intermediate care; those who demand up to ten hours of Nursing assistance, as in high-dependence or semi-intensive care; and patients who demand 18 hours, as in intensive care. In the period under study, the hospitalized women were classified, on average, in the complexity level to high-dependence care measures with presence of women both in minimal and in intensive care. Thus, the units researched presented patients with high demand for care, both in terms of time and care complexity. 

 In Clinical and Surgical Oncology hospitalization units of a reference hospital for the *Triângulo Sul* macro-region that used the Patient Classification System, there was a higher percentage of patients in minimal and intermediate care, although they also include patients in semi-intensive and intensive care ^(^ ^17^ ^)^ . Despite the differences in the assessment instrument used and the participants’ types of cancer when compared to this study, it is possible to point out a higher mean of Nursing hours consistent with patients in high dependence, semi-intensive or intensive care in the Clinical and Surgical Oncology hospitalization units of women in the current study. This can be contextualized by the fact that women demand greater Nursing care beyond physical and therapeutic issues, requiring greater support to also meet the psychological, emotional and self-image aspects ^(^ ^5^ ^)^ , which can indirectly burden the Nursing team. 

 Women’s admission required a mean of twenty-four minutes more Nursing care than on the hospitalization outcome day, in agreement with other studies carried out in intensive care units that also presented higher workloads on admission ^(^ ^23^ ^-^ ^25^ ^)^ . 

 Most of the participants (84.4%) had the outcome of being discharged home, when it is expected that the women are stable and in conditions for self-care or care with the help of a family member. In addition to that, at the time of admission, the patients can be in the acute phase of the disease, requiring immediate interventions, invasive therapeutic measures, collection of tests and complex care, which may be more intense on the first hospitalization day ^(^ ^23^ ^,^ ^25^ ^)^ , in addition to demanding greater attention to the family ^(^ ^25^ ^)^ . Although most of the women in this study were admitted for elective surgical treatments, stable and without disease deterioration, they required significant time from the Nursing team. The aforementioned considering the beginning of surgical preparation, collection of tests and related procedures, in addition to the guidelines for patients and family members on unit routines, clarification of doubts and managerial issues related to hospital admission, justifying a greater workload in women’s admission. 

 The monitoring and control activities, administrative and managerial tasks, hygiene procedures and medications were the ones that had the highest percentage of days scored and also demanded more time from the Nursing team in the 24 care hours for each woman. Similar results were found in a Clinical Oncology unit using a patient classification instrument indicated by the National Resolution for staff sizing ^(^ ^17^ ^)^ . It is interesting to point out that the third item that most demanded Nursing time was related to administrative and managerial activities, being considered an indirect care measure. Care documentation demands considerable time from the Nursing team, being associated with workload in other studies ^(^ ^26^ ^-^ ^27^ ^)^ . 

 The highest demand for care was also displayed by women with metastases, when compared to those without them, according to the univariate analysis. The most prevalent metastases were pulmonary/pleural and bone. Women with metastases also presented lower functional capacity. Tumor metastases can generate limitations and suffering, leading to surveillance and continuous management by the health team ^(^ ^28^ ^)^ , corroborating the results. Among Ethiopian women with breast cancer, the most advanced cancer stage (stage IV with presence of distant metastasis) was related to the following: more intense pain; loss of appetite and symptoms in the arms; having worse quality of life; and, consequently, requiring greater professional attention ^(^ ^29^ ^)^ . 

 In addition, the metastasis diagnosis was found in most of the women in palliative care, which may contribute to a greater demand for care, as these women had the highest mean NAS score. However, in this study there was no significant percentage of participants in this condition. In addition to palliative treatment requiring more care, work overload and the deficit of human resources also exert negative impacts on the transition from exclusive palliative care in women with breast cancer ^(^ ^30^ ^)^ , disqualifying care. 

 There was also a moderate correlation between the mean NAS score and the hospital time in the Oncology units, indicating that longer hospitalization time can be related to higher workloads, although this correlation is not maintained in the multivariate analysis. In the context of this hospital, this correlation can be explained by the fact that women who stay longer in hospital usually have cancer at a more advanced stage ^(^ ^31^ ^)^ or are in worse clinical conditions and, consequently, demand more Nursing care. A similar relationship was signaled in an Australian study which concluded that the implementation of a numerically more balanced nurse-patient relationship policy in medical-surgical units reduced the patients’ hospitalization times ^(^ ^9^ ^)^ . 

 The women that were readmitted during the four months of this study also required more Nursing care than those who were not readmitted, according to the univariate analysis. In general, people readmitted to Oncology hospitalization units present a decline in activities of daily living and lower functional capacity ^(^ ^1^ ^)^ and, therefore, are more dependent on care from the Nursing team. The chance of hospital readmission within seven days after discharge is greater in hospitals with a lower proportion of nurses per number of patients, characterizing a greater workload ^(^ ^9^ ^)^ . 

 The gynecological cancer diagnosis also required more Nursing hours than women with breast cancer, a relationship maintained in the regression model. Lower functional capacity was also observed among the women with gynecological cancer, characterizing a possible need for assistance, when compared to those with breast cancer, which can contribute to the greater need for Nursing hours. Most gynecological cancer diagnoses occur in intermediate and advanced stages of the disease, leading to more serious lesions and more complex and invasive treatments ^(^ ^32^ ^)^ . In cases of cervical cancer, for example, when the diagnosis is early, it is possible to use only surgical methods, which cause fewer harms to physical, social, emotional and functional well-being ^(^ ^33^ ^)^ . This denotes the relevance of early diagnosis in terms of Public Health, as well as staffing of units that consider the epidemiological profile of the women cared for. 

 The type of treatment to which the women were submitted during their hospitalization was also considered as a factor related to the Nursing workload in Oncology units. Thus, the participants undergoing surgical treatments required lower workloads, as they received a mean of one hour and four minutes less Nursing assistance, when compared to those undergoing clinical treatments. All participants who died during the research were undergoing clinical treatments, with patients in the process of death being those who required the greatest workload ^(^ ^23^ ^-^ ^24^ ^)^ . This was also the case with the women in palliative care, as they all received clinical treatments and obtained the highest NAS scores, contributing to this relationship. It is estimated that nurses spend a mean of 20% of their working time on palliative care, associated with professional exhaustion ^(^ ^11^ ^)^ . In these palliative care and death situations, Nursing care focuses not only on the patients, but also on the family members’ needs, interfering with the time devoted to care ^(^ ^34^ ^)^ . 

 Other studies also show that Clinical Oncology units have a higher percentage of patients with high dependency, in semi-intensive and intensive care, when compared to surgical units ^(^ ^17^ ^)^ . Undergoing clinical treatments caused a 20.54-point increase in the NAS score among the cancer patients admitted to an intensive care unit in the COVID-19 pandemic context, when compared to those undergoing surgical treatments ^(^ ^35^ ^)^ . 

 In this research, data collection also took place during the COVID-19 pandemic, with difficulties obtaining beds available in intensive care units, making it possible to contextualize the presence of patients with greater clinical deterioration in the clinical hospitalization unit and, therefore, increasing the Nursing workload. On the other hand, with technological advances, Oncology surgeries have become less invasive, with reduced sequelae and rapid recovery ^(^ ^36^ ^)^ , which also ends up demanding less from the Nursing team. 

 As the functional capacity assessment, according to KPS, identifies clinical decline and dependence in carrying out activities of daily living ^(^ ^18^ ^)^ in the participating women, applying this scale can guide the assessment of the Nursing care complexity during women’s hospitalization, which is relevant, given that patient care complexity is a predictor of Nursing workload ^(^ ^27^ ^)^ . Thus, in this study, according to KPS, the participants’ functional capacity proved to be related to the Nursing workload in this context. 

 A participant who has an admission KPS score of less than or equal to 50%, indicating inability, limited self-care and need for supportive care, represents an increase in the NAS score when compared to those with KPS values above 50%. Although chemotherapy and radiotherapy compromise the patients’ functional capacity, self-care and quality of life ^(^ ^37^ ^-^ ^38^ ^)^ , in this sample, the relationship between lower functional capacity and higher NAS scores could not be adequately investigated regarding these treatments during the hospitalization, as few participants underwent chemotherapy (4.8%) and radiotherapy (2.2%). 

 The KPS score was also related to Nursing workload in a study carried out in an Oncology intensive care unit, although functional capacity was much lower in this specific context: 92.7% of the patients obtained KPS values equal to or less than 30% ^(^ ^23^ ^)^ . Furthermore, the analysis of the dependence level profile of the patients assisted can guide organization of the team, with integration of other health professionals, according to the most representative care demands of the unit ^(^ ^20^ ^)^ and, thus, contribute to interdisciplinary work and reduce Nursing workload. Therefore, multidisciplinary, coordinated and comprehensive work can respond to clinical issues and psychosocial needs faced in the Oncology field ^(^ ^39^ ^)^ . 

 As was the case with a contemporary study ^(^ ^27^ ^)^ , this research did not intend to evaluate the workload for Nursing staff sizing, as this is a strategic action in a public hospital and outside the Nursing care team control. However, the results presented identified the necessary care measures and characteristics of the women with gynecological and/or breast cancer that require longer care times, and can help managers and professionals working in these units to make possible institutional changes, such as optimizing the existing resources, improvements in processes, flows and work environment ^(^ ^27^ ^)^ , depending on the profile of the hospitalized population. 

 These changes can contribute to a favorable and safe work environment, improve workers’ health conditions, improve labor division and interpersonal relationships, increase professional satisfaction and, consequently, improve the quality of the care provided ^(^ ^7^ ^,^ ^12^ ^)^ . 

This study allowed identifying the main differences in the Nursing care demand for hospitalized women with breast and/or gynecological cancer, as well as the sociodemographic and clinical factors that may interfere with the Nursing workload in Oncology inpatient units. The results can qualify management interventions to plan improvements in the quality of the assistance provided to women with breast and/or gynecological cancer, as well as in the work environment, through qualifications in the efficient management of human resources and improvements in work processes and routines. In addition to that, this study was a pioneer in applying the NAS instrument with content adapted to cancer patients, allowing for a more accurate assessment of the Nursing workload in Hospital Oncology units, as well as contributing to expanding its use in the clinical practice.

 The limitations of this study refer to its cross-sectional design, making it impossible to establish a causal link between the results. In addition to that, no studies were found that would allow comparability with the specific patient profile of the data collection institution in this study, especially with regard to differences in the mean NAS score, care time for each Nursing activity and factors related to the workload. The multifactorial nature of the Nursing workload requires an analysis beyond the patients’ needs ^(^ ^20^ ^)^ , as NAS does not accurately measure completeness of the Nursing work demands, despite having been adapted to the context of cancer patients. Although NAS explores some indirect care activities, other issues that may interfere with the workload are not measured, mainly the psychological and emotional burden of professionals working in the Oncology area. Therefore, new studies are suggested to investigate other domains that may be related to the Nursing workload in the care of women with cancer, such as the emotional, personal and institutional components. 

 Finally, the use of instruments that allow measuring the Nursing workload, such as the Patient Classification System and NAS, require commitment from the management to support their application and adequately interpret the data related to workload ^(^ ^20^ ^,^ ^40^ ^)^ . 

## Conclusion

The women with gynecological/ breast cancer hospitalized in inpatient unit, had a mean of seven Nursing care hours a day, according to NAS application, adapted to the Oncology context. The most prevalent activities that required the most time from the Nursing team over the 24 hours for each woman with cancer were monitoring and controls, administrative and managerial tasks, hygiene procedures and drug preparation and administration.

It was also verified that the cancer diagnosis (breast/gynecological), the type of current treatment (clinical or surgical) and the women’s functional capacity at admission, as measured by KPS, were factors related to Nursing workload. The women with breast cancer presented a decrease in the NAS score when compared to those with gynecological cancer, as well as those undergoing surgical treatments showed a decrease in NAS when compared to those subjected to clinical treatments. On the other hand, the women with KPS values less than or equal to 50% at admission, that is, lower functional capacity and less autonomy for self-care, had an increase in their NAS scores when compared to those with KPS scores above 50%.

These findings denote the relevance of instruments for assessing Nursing workload, such as NAS adapted to the Oncology context, in order to offer diverse information that guides Nursing care management and planning in inpatient Oncology units.
